# Binding between Crossveinless-2 and Chordin Von Willebrand Factor Type C Domains Promotes BMP Signaling by Blocking Chordin Activity

**DOI:** 10.1371/journal.pone.0012846

**Published:** 2010-09-23

**Authors:** Jin-Li Zhang, Lucy J. Patterson, Li-Yan Qiu, Daria Graziussi, Walter Sebald, Matthias Hammerschmidt

**Affiliations:** 1 Institute for Developmental Biology, Cologne Biocenter, University of Cologne, Cologne, Germany; 2 Department of Physiological Chemistry II, Biocenter, University of Wuerzburg, Wuerzburg, Germany; 3 Center of Molecular Medicine Cologne (CMMC), University of Cologne, Cologne, Germany; 4 Cologne Excellence Cluster on Cellular Stress Responses in Aging-Associated Diseases (CEDAD), University of Cologne, Cologne, Germany; Max Planck Institute of Molecular Cell Biology and Genetics, Germany

## Abstract

**Background:**

Crossveinless-2 (CV2) is an extracellular BMP modulator protein of the Chordin family, which can either enhance or inhibit BMP activity. CV2 binds to BMP2 via subdomain 1 of the first of its five N-terminal von Willebrand factor type C domains (VWC1). Previous studies showed that this BMP binding is required for the anti-, but not for the pro-BMP effect of CV2. More recently, it was shown that CV2 can also bind to the BMP inhibitor Chordin. However, it remained unclear which domains mediate this binding, and whether it accounts for an anti- or pro-BMP effect.

**Principal Findings:**

Here we report that a composite interface of CV2 consisting of subdomain 2 of VWC1 and of VWC2-4, which are dispensable for BMP binding, binds to the VWC2 domain of Chordin. Functional data obtained in zebrafish embryos indicate that this binding of Chordin is required for CV2's pro-BMP effect, which actually is an anti-Chordin effect and, at least to a large extent, independent of Tolloid-mediated Chordin degradation. We further demonstrate that CV2 mutant versions that per se are incapable of BMP binding can attenuate the Chordin/BMP interaction.

**Conclusions:**

We have physically dissected the anti- and pro-BMP effects of CV2. Its anti-BMP effect is obtained by binding to BMP via subdomain1 of the VWC1 domain, a binding that occurs in competition with Chordin. In contrast, its pro-BMP effect is achieved by direct binding to Chordin via subdomain 2 of VWC1 and VWC2-4. This binding seems to induce conformational changes within the Chordin protein that weaken Chordin's affinity to BMP. We propose that in ternary Chordin-CV2-BMP complexes, both BMP and Chordin are directly associated with CV2, whereas Chordin is pushed away from BMP, ensuring that BMPs can be more easily delivered to their receptors.

## Introduction

Bone morphogenetic proteins (BMPs) play a major role in early dorsal-ventral (D–V) patterning of developing animal embryos, determining differential cell fates along the future D–V axis by a gradient of BMP signaling. In fish and frog embryos, this gradient is generated by the localized secretion of BMP inhibitors Chordin, Noggin, Follistatin, Cerberus and others [1], whereas the *bmp* genes themselves are initially uniformly expressed throughout the entire embryo [2]–[4]. Chordin is a large secreted protein generated in the so-called Spemann-Mangold organizer. It binds directly to BMPs and prevents BMP binding to their cognate receptors, leading to dorsalization of the embryos in overexpression studies [5], [6], [7], while loss of Chordin activity leads to ventralized embryos [8], [9], [10]. The binding of Chordin to BMP is mediated by its von Willebrand factor type C (VWC) domains, also called cystein-rich (CR) repeats [11]. Chordin binds to BMP2 preferentially via its VWC1 and VWC3 domains, and to BMP7 via its VWC1 and VWC4 domains. The VWC2 domain of Chordin, however, was found to be neither necessary nor sufficient for BMP binding [12].

In vivo Chordin functions mainly as a BMP antagonist (see above). But in certain contexts, Chordin can have subtle long-range pro-BMP effects, as best demonstrated for the *Drosophila* Chordin and BMP2/4 homologues Sog and Dpp. Compared to vertebrates, fly embryos display an inverse Dpp gradient, with highest levels dorsally, while Sog is made in ventrolateral domains. Sog/Dpp complexes originating from such ventrolateral regions diffuse towards the dorsal side of the embryo, where Sog is proteolytically cleaved by Tolloid and Dpp is released from the complex, resulting in a Sog-dependent “up-hill” transport of Dpp [11], [13], [14]. A similar mechanism seems to be at play between Chordin and Bmps in zebrafish and Xenopus to bring BMPs into ventral-most domains [2], [15], [16]. Dual contrary effects on BMP signaling have also been shown for Twisted gastrulation (Tsg), a cofactor of Chordin-BMP interaction. Tsg forms a ternary complex with Chordin and BMP, making Chordin a better BMP inhibitor. On the other hand, Tsg facilitates the cleavage and inactivation of Chordin by metalloproteinases of the Tld (Tolloid)/Xld (Xolloid) family. In this context, Tsg behaves as a pro-BMP factor [17]–[20].

Another BMP modulator protein with dual activity is Crossveinless-2 (CV2), a Chordin family member containing five VWC domains at its N-terminus [1], [21]. It is often co-expressed with BMPs, and its expression is positively regulated by BMP signaling [22]–[24]. Upon forced expression in different in vivo and in vitro assays, it can either promote or inhibit BMP signaling in a context- and concentration-dependent manner [22]–[30]. Based on genetic analyses, CV2 was first discovered in *Drosophila* where it is required for signaling by the BMP homologues Dpp and Gbb during the formation of wing crossveins [31]–[33]. In vertebrates, loss-of-function studies revealed an essential role of CV2 to promote BMP signaling during mouse organogenesis [34] and D–V patterning of the gastrulating zebrafish embryo [23], [35], whereas during D–V patterning in Xenopus, its primary function is to block BMPs [22]. Ambrosio et al. also showed that Xenopus CV2 can directly bind Chordin and that a BMP4/CV2/Chordin ternary complex can be formed [22]. This, together with the similar and cooperative effects of Chordin and CV2 upon overexpression in Xenopus embryos, led to the assumption that CV2 generally promotes Chordin's function, resulting either in an anti-BMP effect, or in the Chordin-dependent pro-BMP effect in ventral-most regions (see above), when sequestering Chordin/BMP complexes on the ventral side of the embryo, thereby facilitating ventral diffusion of the complexes, and ensuring that BMPs liberated from Chordin inhibition upon Tolloid-mediated proteolysis generate peak signaling levels [22]. In contrast, data presented here suggest a Chordin-antagonizing function of CV2 to be at least partly responsible for its pro-BMP effect.

Thus far, particular interaction domains within the CV2 protein have only been characterized for its binding to BMPs, but not to Chordin. Structural analysis revealed that subdomain 1 (SD1) of the first VWC domain (VWC1) of CV2 is involved in BMP binding, whereas SD2 of VWC1, the other four VWCs and the C-terminal domains of CV2 do not participate in binding [12], [30]. Accordingly, the in vivo anti-BMP activity of CV2 is confined to the VWC1 domain, since overexpression of a BMP binding-deficient CV2 mutant with a point mutation in VWC1 lost the anti-BMP activity [12], [30]. However, the pro-BMP function of the mutant CV2 version was retained, suggesting that it is independent of BMP binding, and that the anti- and pro-BMP activities can be structurally separated. However, the domains accounting for the pro-BMP activity of CV2 remained unknown.

In this report we show that the binding site of Chordin for CV2 is restricted to its VWC2 domain, while the binding site of CV2 for Chordin is a composite interface formed by the SD2 of VWC1 and VWC domains 2, 3 and 4. In vivo functional analysis in zebrafish demonstrates that Chordin binding of CV2 correlates with its pro-BMP activity in a largely Tolloid-independent manner. Possible mechanisms are discussed via which CV2 might promote BMP signaling by inhibiting Chordin's anti-BMP activity.

## Results

### Binding of CV2 to Chordin

**Figure 1:**
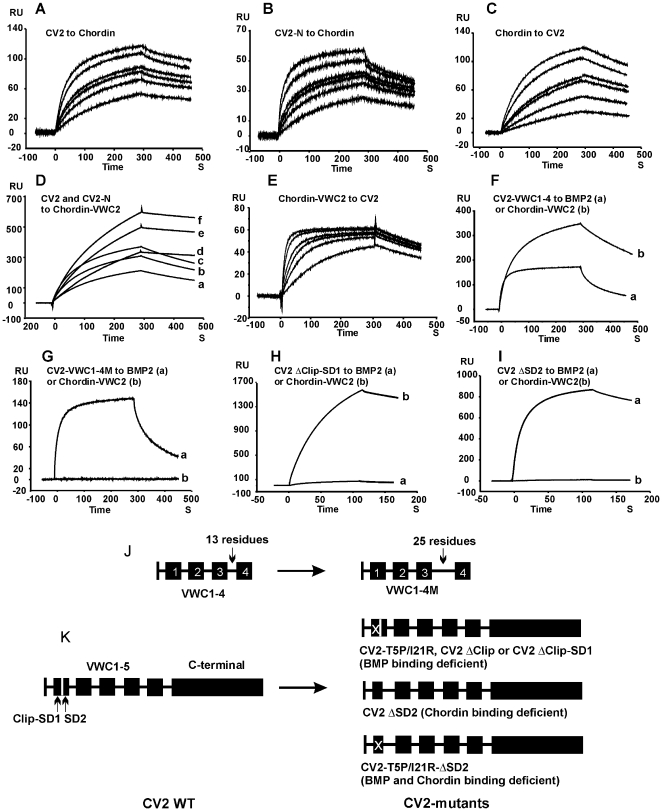
BIAcore analysis of CV2/Chordin interaction. Sensograms showing the binding of 100,200,300,400,800 and 1200 nM CV2 (A) or CV2-N (B) to immobilized Chordin; 50, 100, 150, 200, 400 and 600 nM Chordin to immobilized CV2 (C); 100, 200 and 300 nM CV2-N (D, a,b,c) or CV2 (D,d,e,f) to immobilized VWC2 of Chordin; 10, 20, 30, 40, 80, 120 nM VWC2 of Chordin to immobilized CV2-N (E); 200 nM CV2-VWC1-4 (F) or CV2-VWC1-4M (G) to immobilized BMP2 (Fa, G,a) or VWC2 of Chordin (Fb, G,b); 300 nM CV2 ΔClip-SD1(H) or CV2 ΔSD2 (I) to BMP2 (Ha, Ia) or VWC2 of Chordin (Hb, Ib). (J) and (K) show schemes of the CV2 mutant constructs. Abbreviation: RU, resonance unit.

Previous studies have indicated that CV2 fulfills part of its pro-BMP function via an inhibitory effect on Chordin [23]. To gain insights into the molecular mechanisms, we tested the binding of CV2 to Chordin by BIAcore surface plasmon resonance analysis. CV2 binds immobilized Chordin with an apparent *K*_D_ of 175 nM (Figure 1A and Table 1). Similar result was obtained when the CV2 protein was immobilized on the chip (Figure 1C and data not shown). Interestingly, similar to the binding of CV2 to BMP2 [12], the CV2-Chordin binding occurs via the N-terminal VWC repeats of CV2, as the CV2-N protein consisting of VWC1-5 binds to Chordin with the same affinity as full-length CV2 (Figure 1B and Table 1).

**Table 1:** Binding of CV2 and CV2 fragments to Chordin, to VWC2 domain of Chordin, and to BMP2.

	Chordin	Chordin	BMP2
		VWC2	
CV2 Proteins		Apparent *K*_D_	
		(nM)	
CV2	175	150	22
CV2-VWC1-5	170	140	25
CV2-VWC1-4	180	145	21
CV2-VWC1-3	NB	NB	24
CV2-VWC2-4	NB	NB	NB
CV2-VWC3-4	NB	NB	NB
CV2-VWC4-5	NB	NB	NB
CV2-VWC1-4M	NB	NB	23
CV2 ΔClip-SD1	188	170	NB
CV2 ΔSD2	NB	NB	22
CV2 T5P/I21R	183	172	18000
CV2 ΔClip	178	180	50000

NB: no binding.

### Chordin VWC2 is responsible for CV2 binding, whereas a composite interface of VWC 1,2,3 and 4 of CV2 is involved in Chordin binding

Chordin and CV2 are large proteins consisting of multiple VWC domains. To determine which of them are involved in Chordin/CV2 binding, we expressed different Chordin and CV2 fragments and analyzed their mutual binding by BIAcore surface plasmon resonance. We found that CV2 and CV2-N bound to the immobilized VWC2 domain of Chordin with an affinity similar to that between CV2/CV2-N and full-length Chordin (Figure 1D and Table 1). No binding was found between CV2 and the other three Chordin VWC domains 1, 3 and 4 (data not shown). These results indicate that of the four VWC domains of Chordin, VWC2 is the only that binds to CV2. Interestingly, the three other VWC domains of Chordin, but not VWC2, are involved in its binding to BMP and Tsg [12], indicating that different VWC domains of Chordin account for binding to BMP versus CV2. Noteworthy, when CV2 was immobilized on the BIAcore chip and Chordin VWC2 was applied in solution, an apparent *K*_D_ of 5 nM was obtained (Figure 1E), compared to an over 30-fold higher *K*_D_ value (30 times lower affinity) in the reverse experimental set-up (see above). This difference is due to different association rates, whereas the dissociation rates were the same for both set-ups (compare Figure 1 D and E). One explanation could be the smaller size and correspondingly faster association kinetics of soluble Chordin VWC2 versus soluble CV2.

To dissect the Chordin-binding domains within CV2, we expressed CV2 fragments consisting of different VWC domains, namely CV2-VWC1-5 (CV2-N), CV2-VWC1-4, CV2-VWC1-3, CV2-VWC2-4, CV2-VWC3-4 and CV2-VWC4-5, and determined their affinities to full-length Chordin or its VWC2 domain. In parallel, the affinities of the fragments to BMP2 were measured. As shown in Table 1, and consistent with previous results [12], [30], the CV2 fragments VWC1-5, VWC1-4 and VWC1-3, which contain VWC1, could bind to BMP2 with the same affinity as full-length CV2 and VWC1 alone. In contrast, only VWC1-4 bound Chordin with an affinity similar to full-length CV2 and VWC1-5, whereas VWC1-3 did not show any binding. Furthermore, fragments VWC2-4, VWC3-4 and VWC4-5 neither bound Chordin nor BMP2. These results indicate that VWC domains 1 and 4 of CV2 are absolutely necessary for Chordin binding, whereas VWC5 is dispensable. It is still unknown whether VWC1 and VWC4 are sufficient for Chordin binding, or whether VWC2 and VWC3 are required as well, since no constructs lacking or replacing these internal domains have been tested as yet.

It is likely that the VWC4 domain forms a composite interface with VWC1 or VWC1-3 for Chordin binding. To test this idea, we generated a VWC1-4 insertion mutant (VWC1-4M), in which 12 random residues GSLVPRGSMLSG were inserted between VWC3 and VWC4 (Figure 1J). VWC1-4M could no longer bind Chordin, whereas its BMP binding activity was retained (Figure 1F,G and Table 1), indicating that the composite interface of CV2 for Chordin could be destroyed by enlarging the distance between VWC4 and VWC1-3.

Our results obtained thus far suggest that in contrast to the strict separation of the different VWC domains of Chordin for binding to BMP2 versus CV2 (see above), the VWC1 domain of CV2 is involved in both BMP2 and Chordin binding. It is known that the VWC1 domain of CV2 consists of three parts: the N-terminal segment called Clip, and subdomains SD1 and SD2 (Figure 1K). The Clip and SD1 are responsible for BMP2 binding, whereas SD2 is dispensable for this binding [12], [30]. To determine which part of CV2-VWC1 is involved in Chordin binding, different subdomain truncation mutants (Figure 1K) were generated and analyzed by BIAcore surface plasmon resonance. CV2 ΔClip-SD1 that lacks the Clip and SD1 failed to bind BMP2, but bound Chordin with normal affinity (Figure 1H and Table 1). In contrast, the mutant CV2 ΔSD2, in which SD2 of VWC1 is missing, could bind to BMP2 normally, whereas binding to Chordin was lost (Figure 1I and Table 1). This suggests that within the CV2-VWC1 domain, Clip-SD1 is involved in BMP binding and SD2 in Chordin binding.

In summary, our biochemical analysis demonstrates that the VWC2 domain of Chordin, which is dispensable for binding to BMP2 and Tsg, is involved in CV2 binding, while a composite interface of CV2 consisting of SD2 of VWC1 and VWC2, 3, 4, all of which are dispensable for BMP binding, is responsible for Chordin binding.

### Binding of CV2 to Chordin promotes BMP signaling in vivo

**Figure 2:**
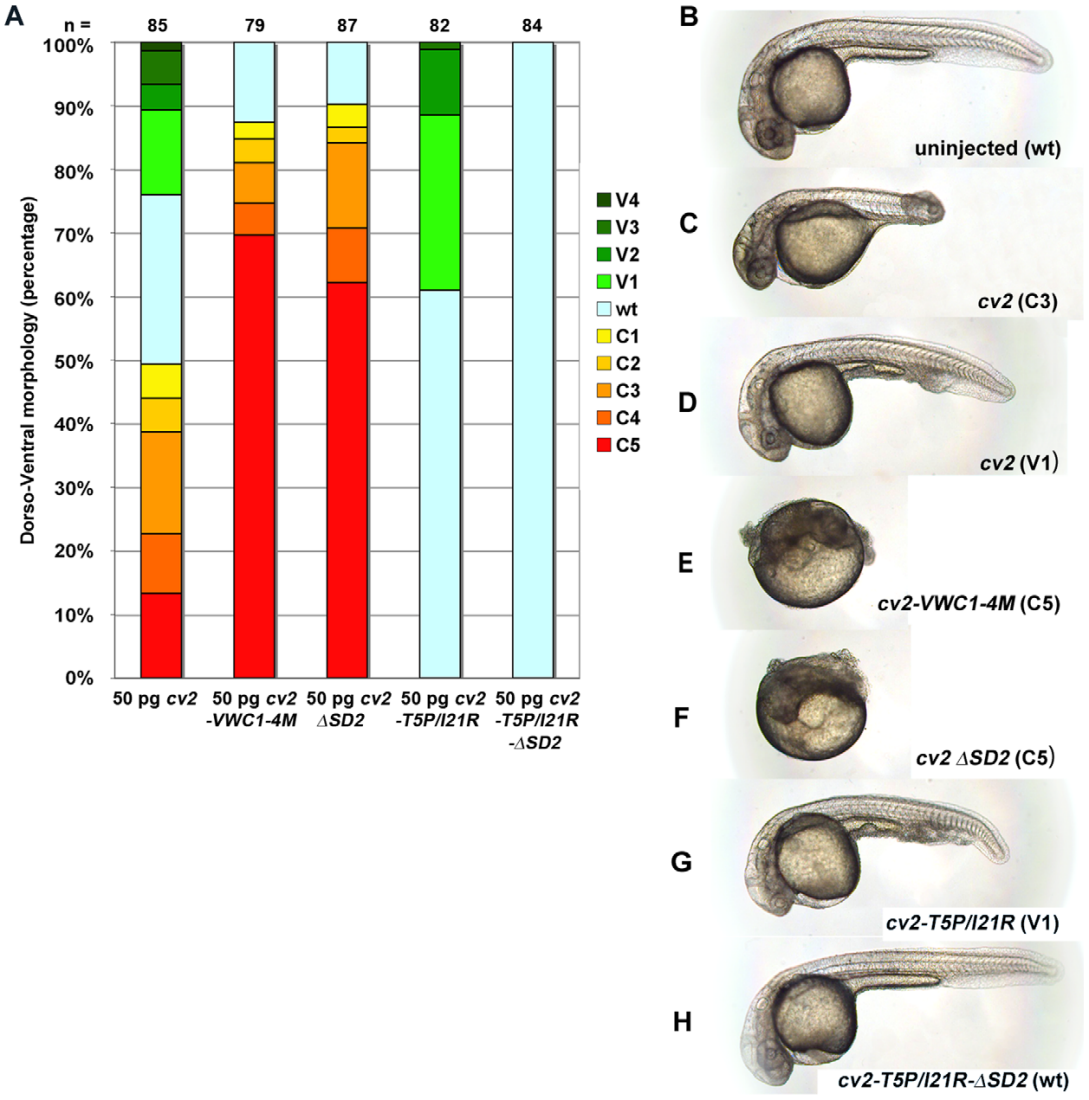
Binding of CV2 to Chordin promotes BMP signaling in vivo. (A) Graphical illustration of proportions of phenotypes generated upon injection of different CV2 constructs. For classification from C5 (strong dorsalization) to V4 (strong ventralization), see Mullins et al [47] and Kishimoto et al (38). Numbers of analyzed embryos are indicated above the columns. (B–H) Representative zebrafish embryos after mRNA injections as in (A). Lateral views of live embryos at 32 hpf.

To test the effect of CV2/Chordin binding in vivo, we performed mRNA injection experiments in zebrafish embryos. Consistent with our previous results [23], [30], overexpression of wild-type CV2 led to moderate dorsalization, indicative of reduced BMP signaling (Figure 2A,C), or weak ventralization, indicative of enhanced BMP signaling (Figure 2A,D). Injection of mRNA of the Chordin binding-deficient mutant versions *cv2-VWC1-4M* and *cv2-ΔSD2* resulted in strongly dorsalized embryos (Figure 2E, F), while no ventralized embryos were obtained. This suggests that the pro-BMP effect of CV2 requires Chordin/CV2 binding, whereas its anti-BMP effect is Chordin-independent.

We next tested the in vivo effect of a compound CV2 mutant version that lacks both BMP and Chordin binding. We have previously shown that a CV2-I21R mutant, which bears an amino acid exchange in the SD1 domain of VWC1 and which has 40-fold lower affinity to BMP2, lost its anti-BMP activity but retained the pro-BMP activity, as revealed by the absence of dorsalized, but the presence of ventralized embryos upon mRNA injections [30]. A double mutant CV2-T5P/I21R with an additional point mutation in the Clip and a 1000-fold lower BMP affinity (Table 1) showed similar ventralizing effects when injected into zebrafish embryos (Figure 2A,G). However, when the ΔSD2 mutation was added, the triple mutant CV2-T5P/I21R-ΔSD2 (Figure 1K) lost both the pro- and anti-BMP activities, and had no effect of D–V patterning at all (Figure 2A,H). Together, this indicates that, linked to BMP- versus Chordin-binding, the anti- versus pro-BMP in vivo effects of CV2 can be structurally separated.

### The pro-BMP activity of CV2-T5P/I21R requires the presence of Chordin

**Figure 3:**
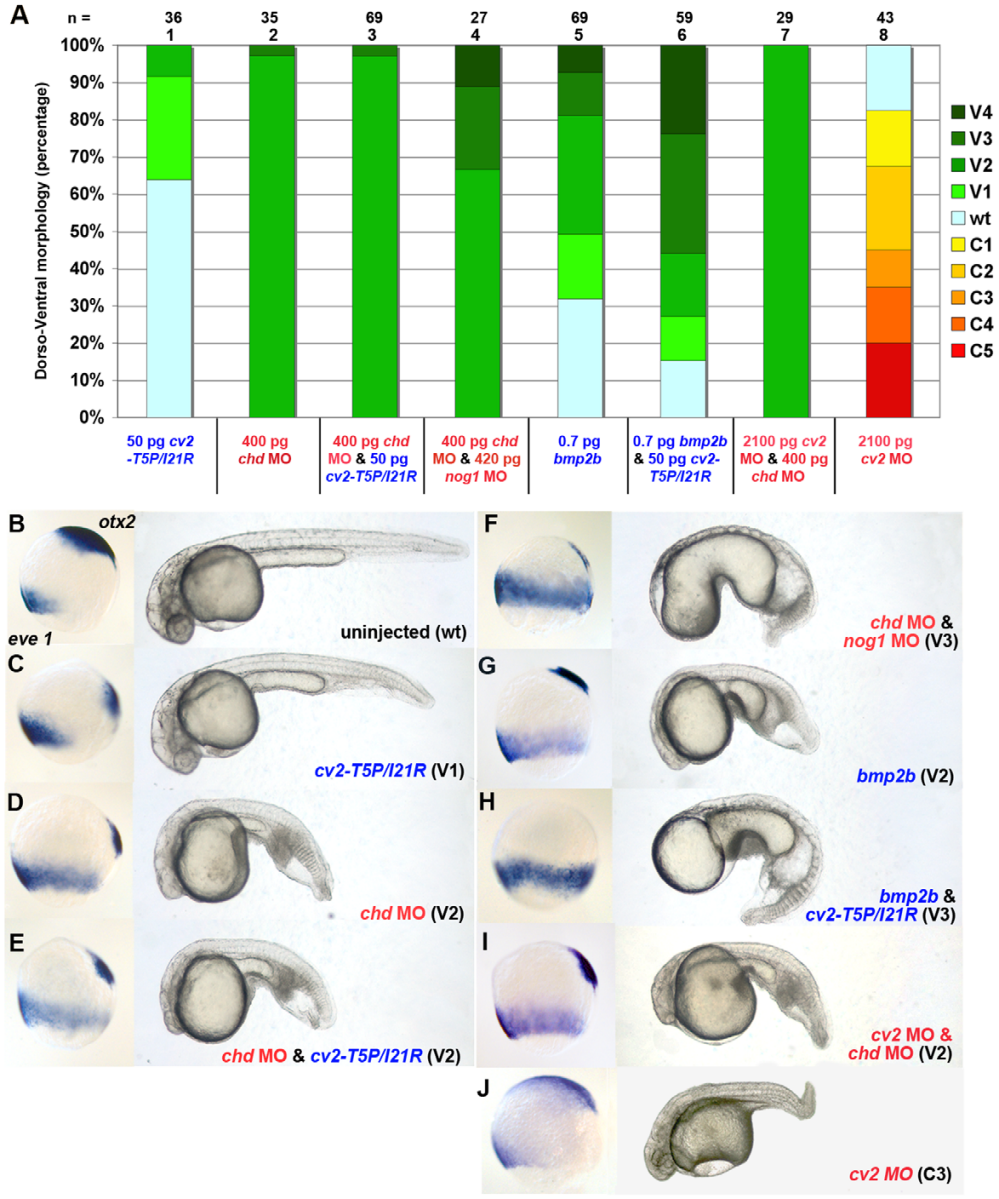
The pro-BMP activity of CV2-T5P/I21R requires the presence of Chordin. (A) Graphical illustration of proportions of dorsalized and ventralized phenotypes generated upon injection of *cv2-T5P/I21R* or *bmp2b* mRNA (in blue letters), *cv2*, *chd* or *nog1* MOs (in red letters), or mixtures of the mRNA/MOs in different combinations. Numbers of analyzed embryos are indicated above the columns; columns are numbered. (B–J) Representative zebrafish embryos after mRNA and/or MO injection as in (A). Right panels show overall morphology of live embryos in lateral views at 32 hpf, left panels show embryos at 80% epiboly (mid gastrula stage), after whole mount *otx2*/*eve1* double in situ hybridizations (domains indicated in B), lateral views, dorsal to the right. The expression of the dorsal marker *otx2* is reduced in ventralized and expanded in dorsalized embryos, whereas the ventral marker *eve1* shows contrary shifts.

**Figure 4:**
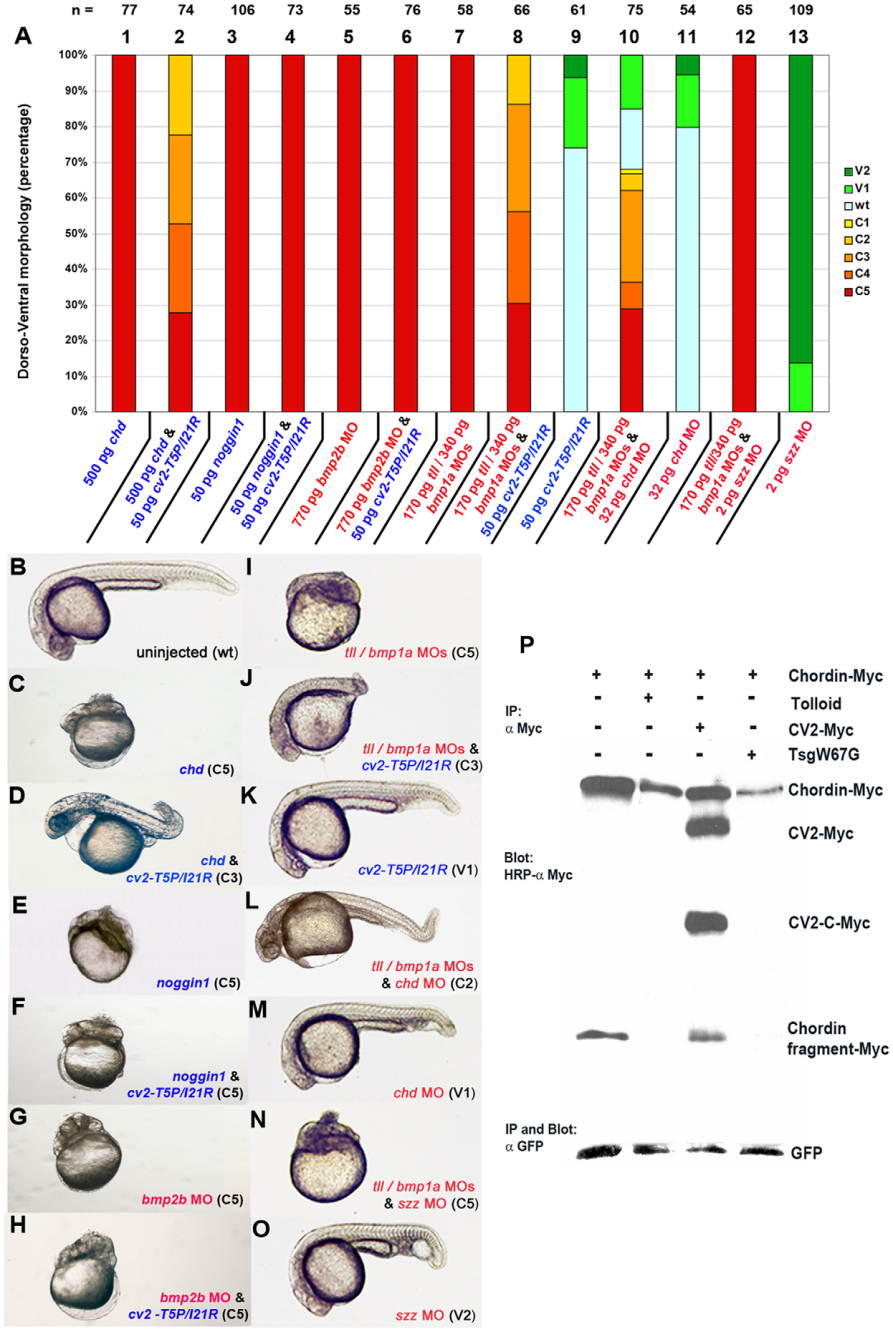
CV2 can antagonize the dorsalizing effect of Chordin, but not of Noggin1, while the anti-Chordin effect of CV2 is both dependent and independent of Tolloid/Bmp1a. (A) Graphical illustration of proportions of dorsalized and ventralized phenotypes generated upon injection of *cv2-T5P/I21R* mRNA into wild-type embryos (lane 9), into embryos after *chordin* or *noggin1* overexpression (lanes 1–4), or into *bmp2b* (lanes 5,6) or *tolloid*/*bmp1a* morphants (lanes 7,8), in comparison to the response of the *tolloid*/*bmp1a* double morphants to co-injection of *chordin* MO (lanes 10,11) or *sizzled* MO (lanes 12,13). Numbers of analyzed embryos are indicated above the columns; columns are numbered. (B–O) Representative zebrafish embryos after mRNA and/or MO injections as in (A). Lateral views on live embryos at 32 hpf. (P) Western blot detecting C-terminally-Myc-tagged Chordin (upper panel) or GFP (lower panel) in zebrafish embryos after mRNA injections. Lanes were loaded with precipitates of extracts from 50 mid-gastrula stage embryos (85% epiboly stage; 9 hpf) that had been injected at the 1-cell stage with 300 pg *chordin* and – as injection control - *gfp* mRNA per embryo, or with the same amounts of *chordin* and *gfp* mRNA, plus 600 pg mRNA encoding CV2, Tolloid or TsgW67G, respectively. Sibling embryos from the various injections had been allowed to develop further and displayed dorsalization of similar strengths at 32 hpf. In the upper blot, in addition to Chordin, Myc-tagged full-length CV2 and its C-terminal fragment CV2-C could be detected.

To further analyze the functional interaction of CV2 with Chordin, we studied to which extent the pro-BMP effect of CV2 depends on Chordin. For this purpose, we injected mRNA encoding mutant CV2-T5P/I21R that is incapable of binding BMPs into Chordin-depleted embryos. In wild-type embryos, CV2-T5P/I21R had a weakly ventralizing effect (V1–V2; see above and Figure 3A, column 1; Figure 3C). Similarly, Chordin depletion by MO injection produced moderately ventralized embryos (V2; Figure 3A, column 2; Figure 3D). However, co-injection of *chordin* MO with *cv2-T5P/I21R* mRNA did not increase the severity of ventralization, as judged both morphologically and by marker expression criteria (compare Figure 3E with Figure 3D; Figure 3A, columns 2,3). In contrast and as a control, concomitant depletion of Noggin1, a Chordin-independent BMP antagonist, did significantly enhance the ventralization of *chordin* morphants from V2 to V4 (compare Figure 3F with Figure 3D; Figure 3A, columns 3,4), although *noggin1* single morphants were not even weakly ventralized (data not shown; compare with Ref. 45). Furthermore, *cv2-T5P/I21R* mRNA was able to further ventralize V2 class embryos generated by injection of low level *bmp2b* mRNA, in which Chordin is still present (compare Figure 3H with Figure 3G; Figure 3A, columns 5,6). Together, this indicates that the pro-BMP effect of mutated CV2 protein that can bind Chordin, but not BMPs, requires the presence of Chordin. These results are consistent with our previous findings obtained upon concomitant loss of CV2 and Chordin, injecting *cv2* MO into *chordin* mutants [23], which we could confirm by co-injection of *cv2* and *chordin* MO: while injection of *cv2* MO led to moderate dorsalization (Figure 3J; Figure 3A, column 8), indicating that in zebrafish embryos, CV2 has predominant pro-BMP activity, *cv2* MOs failed to alleviate the ventralization of *chordin* morphants (compare Figure 3I with Figures 3D; Figure 3A, columns 2,7), indicating that Chordin is epistatic to CV2, and that CV2 acts via Chordin. A specific pro-BMP effect of CV2 via Chordin-inhibition is further revealed by the ability of CV2-T5P/I21R to alleviate the dorsalization caused by Chordin overexpression. Thus, while embryos solely injected with *chordin* mRNA all displayed strongest dorsalization (C5), the dorsalization in embryos co-injected with *chordin* and *cv2-T5P/I21R* mRNA was much weaker, down to C2 (compare Figures 4C,D,K; Figure 4A, columns 1,2,9). In contrast, *cv2-T5P/I21R* mRNA was totally unable to alleviate the C5 dorsalization caused by injection of *noggin1* mRNA or *bmp2b* MO (compare Figures 4F,H with Figures 4E,G; Figure 4A, columns 3–6). Taken together, these three data sets suggest that CV2 promotes BMP signaling by antagonizing Chordin, which means that its pro-BMP effect is actually an anti-Chordin effect.

### The anti-Chordin effect of CV2 is partly dependent, and partly independent of Tolloid/Bmp1a

The dual, BMP- and Chordin-antagonizing activity of CV2 is functionally reminiscent of Twisted gastrulation, although the two proteins are not structurally related. It is known that Xenopus Tsg facilitates Chordin cleavage by Tolloid proteases, resulting in release of BMP and stimulation of BMP signaling [3], [18]. A similar mechanism has been proposed for CV2 [22]. To test whether CV2 promotes BMP signaling/blocks Chordin activity in a Tolloid-dependent fashion, we studied the effect of CV2-T5P/I21R in zebrafish embryos lacking Tolloid activity. Consistent with previous results [36], zebrafish embryos injected with MOs against Tolloid and the related protease Bmp1a displayed strong dorsalization (Figure 4I; Figure 4A, column 7). This dorsalization could be significantly alleviated upon co-injection of mRNA encoding CV2-T5P/I21R, the mutant version of CV2 capable of binding Chordin, but incapable of binding BMPs (compare Figures 4I,J,K; Figure 4A, columns 7,8,9). In contrast, *cv2-T5P/I21R mRNA* was completely unable to alleviate the dorsalization caused by loss of BMPs themselves (compare Figure 4H with Figure 4G; Figure 4A, columns 5,6), while depletion of Sizzled, which normally enhances the anti-BMP activity of Chordin by inhibiting Tolloid [36], was ineffective in *tll/bmp1a* morphant embryos (compare Figures 4I,N,O; Figure 4A, columns 7,12,13). This indicates that CV2 can block Chordin activity in the absence and independently of Tolloid proteases. However, compared with the effect of MO-mediated knockdown of Chordin itself, the ventralizing activity of CV2-T5P/I21R in Tolloid/Bmp1a-deficient embryos was weaker than in wild-type embryos. Thus, amounts of *cv2-T5P/I21R mRNA* causing V1/V2 ventralization in wild-type embryos only alleviated the strong C5 dorsalization of *tolloid*/*bmp1a* morphants down to C2 (Figure 4J,K; Figure 4A, columns 8,9), whereas *chordin* MOs with an identical V1/V2 ventralizing effect in wild-type embryos could even convert the phenotype of *tolloid/bmp1a* morphants from strongest dorsalization (C5) to mild ventralization (V1) (Figure 4L,M; Figure 4A, columns 10,11). Together, this suggests that CV2 can block Chordin activity both in a Tolloid-dependent and a Tolloid-independent manner.

To examine Chordin proteolysis more directly, we also performed anti-Myc immunoblotting of extracts from zebrafish embryos injected with mRNA encoding Myc-tagged Chordin together with *cv2*, *tolloid* or *tsgW67G* mRNA. The latter encodes a mutant version of Xenopus Tsg that enhances BMP signaling by promoting Tolloid-dependent Chordin degradation [18]. As shown in Figure 4P, compared to embryos injected with *chordin* mRNA only, co-injection of *cv2* mRNA led to a very moderate reduction in the levels of full-length Chordin and Chordin fragments, whereas much more strongly reduced Chordin levels were obtained after co-injection of *chordin* with *tolloid* or *tsg*W67G mRNA. This indicates that, consistent with the phenotypic analyses described above, CV2 inhibition of Chordin occurs both dependently and independently of Tolloid-mediated proteolysis.

### Binding of CV2 to Chordin attenuates Chordin/BMP2 interaction

**Figure 5:**
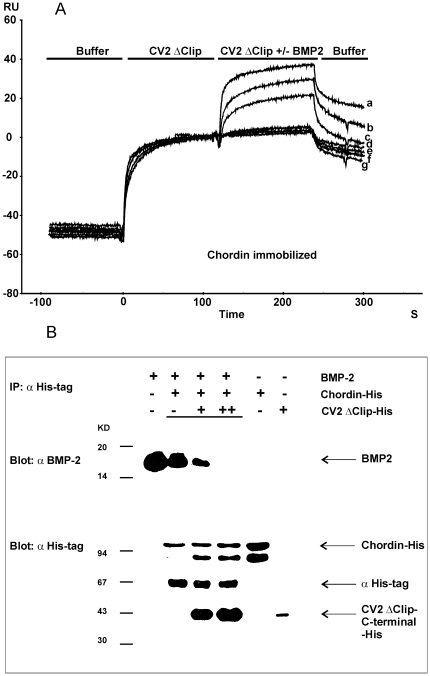
Inhibition of Chordin/BMP2 binding by CV2 ΔClip mutant. (A) BIAcore analysis. 500, 1000 and 3000 nM CV2 ΔClip were first perfused over Chordin immobilized on the BIAcore Chip for 120 seconds, in the second phase the same concentration of CV2 ΔClip (e,f,g, as a negative control) or CV2 ΔClip plus 100 nM BMP2 (b,c,d) were perfused. For a positive control, binding of 100 nM BMP2 alone to immobilized Chordin in a separate experiment is overlaid (a). (B) Co-immunoprecipitation. Mixtures of BMP2, His-tagged Chordin and different concentrations of His-tagged CV2 ΔClip were co-immunoprecipitated by anti-His-tag antibody and Protein-A sepharose (lanes 2–4), followed by Western blotting to detect BMP2 (upper panel) or His-tagged proteins (lower panel) with anti-BMP2 and anti-His-tag antibodies, respectively. Lanes 1, 5 and 6 are loading controls. The CV2 ΔClip protein was auto-catalytically processed [27] into N-and C-terminal fragments, which were associated by disulfide bonds and separated in the reducing SDS gel. Only the His-tagged C-terminal fragment was detected in the Western blot. Abbreviation: RU, resonance unit.

It is known that both CV2 and Chordin bind the BMP2 knuckle epitope for type II receptor via their VWC domains [12], suggesting that the two modulator proteins may compete with each other for BMP binding [23]. Seemingly contradictory, it has been shown recently that CV2, Chordin and BMP-4 can form a ternary complex [22]. As shown above, Chordin and CV2 can bind each other independently of their respective BMP binding sites. However, it remained unclear how BMP is bound in the ternary complex, via CV2, via Chordin, or via both, and whether binding to one of the partners might affect their affinities to the other. To look into this, we studied BMP2/Chordin binding in the presence of BMP-binding deficient CV2 mutants CV2-T5P/I21R or CV2 ΔClip [30], performing surface plasmon resonance analysis (Figure 5A) and co-immunoprecipitations (Figure 5B). Both approaches showed that the CV2 mutants could inhibit BMP2/Chordin interaction (CV2 ΔClip in Figure 5, CV2-T5P/I21R not shown). The reason for the requirement of rather high concentrations of the CV2 mutants to break the Chordin/BMP2 complexes might be the 9-fold different affinity between Chordin and BMP2 (*K*_D_ = 20 nM) [12] versus Chordin and CV2 (*K*_D_ = 180 nM) (Table 1). We conclude that although the binding sites of Chordin for CV2 (VWC2) and BMP2 (VWC1 and 3) are different, and although there is no direct competition between CV2 ΔClip and Chordin for BMP2 binding, the physical interaction between the CV2 mutant and Chordin seems to lower the affinity of the latter for BMP2, possible by inducing some conformational changes within the Chordin protein that affect its BMP binding sites. For the wild-type CV2 protein, this means that it can antagonize Chordin two-fold: first, by the previously revealed direct competition of CV2-VWC1 with Chordin VWC1 or VWC3 for BMP binding [23], and second, by the CV2-induced conformational change of Chordin shown here, ensuring that Chordin is completely excluded from BMP2, even though it may still be associated with CV2 and part of the ternary complex.

## Discussion

### Different VWC domains of CV2 and Chordin contribute to the binding to each other and to BMPs

It has recently been shown that Chordin and CV2 can bind to each other, while the exact domains accounting for this binding remained to be identified [22]. In this study we have analyzed in detail the structural basis of the physical interaction between CV2 and Chordin (Figure 1). Chordin's binding site for CV2 is localized in its VWC2 domain, the only VWC domain that is not involved in the binding of BMP and Tsg [12]. In contrast, CV2's binding site for Chordin is a composite interface formed by SD2 of VWC1, which is not involved in BMP binding [30], VWC4 and probably also VWC2 and 3. This indicates that Chordin's and CV2's binding sites for each other are structurally separated from their BMP binding sites, even though they are all located within the VWC domains. Remarkably, VWC1 of CV2, which consists of a total of 66 amino acids, can be subdivided into two subdomains, SD1 and SD2, which contribute to the binding interaction with BMPs or Chordin, respectively. Thus, the BMP and Chordin binding domains of CV2 are separate, although very close to each other. As the SD1/SD2 subdomain architecture might be a common property of many VWC domains [30], [37], it will for the future be interesting to investigate which subdomains of the non-BMP binding VWC domains of CV2 (2,3 and 4) are involved in Chordin binding. Our results underline the versatile property of VWC domains: they do not only bind to BMP and Tsg, but also to other VWCs. Future studies will have to show whether additional VWC domain-containing proteins involved in BMP/TGF-β binding, such as Kielin-like proteins [38], [39], can bind each other and thus provide further fine-tuning in the regulation of BMP signaling.

### CV2 promotes BMP signaling in vivo by blocking Chordin activity

CV2 has been shown to exhibit both pro- and anti-BMP effects. This is also true for zebrafish. While *cv2* morphants are dorsalized, pointing to a predominant requirement of CV2 to promote BMP signaling, overexpression can cause both dorsalization, indicative of an anti-BMP effect, and ventralization, indicative of a pro-BMP effect. Our in vivo functional analyses with different mutant versions of CV2 lacking either BMP or Chordin binding indicate that the (dorsalizing) anti-BMP effect of CV2 requires BMP binding, whereas its (ventralizing) pro-BMP effect requires Chordin binding (Figure 2). Consistently, we found that the ventralizing effect of CV2 requires the presence of Chordin and that in double-deficient embryos, Chordin is epistatic to CV2: double-deficient embryos display enhanced BMP signaling and are ventralized like chordin single mutants (Figure 3 and [23]). Furthermore, a CV2 mutant version that can only bind Chordin, but not BMP, was able to alleviate the dorsalization caused by overexpression of Chordin, but not the dorsalization caused by overexpression of Noggin1, another extracellular BMP inhibitor (Figure 4). Together, this means that CV2 fulfills its pro-BMP effect by inhibiting Chordin. A similar Chordin-antagonizing effect of CV2 has also been revealed in Xenopus embryos, where loss of CV2 function leads to hypersensitivity for the anti-BMP effect of Chordin [22].

A crucial question is how Chordin-binding of CV2 leads to a pro-BMP effect. Twisted gastrulation (Tsg), another dual modulator of BMP activity that like CV2 can form ternary Tsg/Chordin/BMP complexes, achieves its pro-BMP/anti-Chordin effect by promoting Chordin's degradation through Tolloid proteases [18]. Our data indicate that this also seems to be true to some extent for the anti-Chordin effect of CV2 during dorsoventral patterning of the zebrafish embryo. However, it is clearly not the only mode by which CV2 fulfills its pro-BMP effect, as indicated by its ability to ventralize the zebrafish embryo in the complete absence of Tolloid/Bmp1 activity, and by the rather unaltered levels of exogenous Chordin protein in zebrafish embryos after forced expression of CV2 (Figure 4). Alternatively to Tolloid-dependent proteolysis, extracellular Chordin levels could be down-regulated through endocytosis, consistent with recent work revealing that CV2 can promote Chordin endocytosis upon formation of CV2/Chordin binary complexes in cell culture systems [28]. However, in these systems, CV2 also enhances endocytosis and lysosomal degradation of receptor-bound BMP, which should yield in an opposite, anti-BMP effect [28]. Furthermore, internalization of all three components in Chordin/CV2/BMP ternary complexes is significantly weaker [28]. In zebrafish embryos, CV2 and BMP form a gradient with from dorsal-to-ventral progressively increasing levels, whereas Chordin forms an inverse gradient. Therefore, binary CV2/Chordin complexes should be relatively rare, while ternary Chordin/CV2/BMP complexes should be preferentially formed in lateral regions of the embryo. But what is the activity of BMPs in such extracellular Chordin/CV2/BMP complexes, and how is it modulated by CV2? Pioneering work by Piccolo et al has shown that Chordin can inhibit BMP signaling by blocking the binding of BMPs to their cognate receptors [6], whereas according to a recent report, CV2 can directly bind BMP receptors and can form tripartite complexes with BMPs and type I receptors, thereby promoting BMP signaling when present at low concentrations [24]. According to the data presented in Figure 5, it should be this pro-BMP effect of CV2 that dominates within Chordin/CV2/BMP complexes. Thus, two different mutant versions of CV2 that are incapable of BMP2 binding could, by binding to Chordin, inhibit the binding between Chordin and BMP2. This suggests that within the ternary complex, CV2 may induce a conformational change of Chordin that weakens Chordin's BMP binding capability. Together with the competition of the VWC1 domain of CV2 with the VWC1 or VWC3 domain of Chordin for binding the knuckle epitope of BMP2 used to bind type II BMP receptors [12], this CV2-induced modulation of Chordin's affinity to BMP2 should ensure that within the ternary complex, both BMP2 and Chordin are directly associated with CV2, whereas the physical interaction between Chordin and BMPs is much weaker or completely absent. As the binding affinities of CV2 and BMP receptor Ia for BMP2 are similar [12], the BMP dimer could be delivered to its cognate receptors by CV2, resulting in a pro-BMP activity. It remains to be studied whether in reverse, Chordin/CV2 binding also modulates the affinity between CV2 and BMPs.

### Different modes and effects of Chordin/CV2 interaction might occur along the dorsoventral axis of the fish and frog embryo

Previous work has suggested that Xenopus CV2 might fulfill its pro-BMP effect by concentrating BMP/Chordin complexes on the ventral side of the embryo, where Chordin is cleaved by Tolloid proteases and biologically active BMP is released from the complex [22]. In this case, Chordin and CV2 cooperate in a positive manner, with both Chordin and CV2 displaying a pro-BMP effect. This might be a specialized mechanism, taking advantage of the ventral Chordin sink to bring BMPs up-hill their gradient, e.g. into ventral-most positions of zebrafish and Xenopus embryos, to ensure maximal BMP signaling levels (see also Introduction). The other mechanism of BMP promotion unraveled in this report is crucially different. Here, CV2 is an antagonist, rather than a partner of Chordin, and acts independently of Tolloid function. Such a mechanism might be preferentially at play in more lateral positions of the embryo, lowering the affinity between Chordin and BMPs in ternary Chordin/CV2/BMP complexes (see above). Finally, there seems to be a third mode of Chordin/CV2 interaction, which possibly occurs in more dorsal positions of gastrulating fish and frog embryos and during other developmental processes, with a positive cooperation of the two to block BMP activity. Such a role would be consistent with the effects obtained by CV2 overexpression during Xenopus [22] and zebrafish [23] dorsoventral patterning, and consistent with some of the effects obtained upon morpholino-induced CV2 inactivation in frog [22]. The different net effects along the dorsoventral axis are most likely due to differences in the relative local concentrations of Chordin, CV2 and BMPs, and corresponding differences in the relative levels of the different binary and ternary complexes of the three components [24], as well as differences in the local concentrations of other players such as Tsg and Tolloid/BMP1.

In conclusion, our analyses have unraveled a novel aspect of CV2 action and CV2/Chordin interaction, adding to the complexity of the system fine-tuning the activity of BMP signaling along the dorsoventral axis of the vertebrate embryo.

## Materials and Methods

### Protein expression and purification

Mouse Chordin and its VWC domains, zebrafish CV2, its fragments and mutant versions were expressed in SF9 cells and purified as described [12]. Human BMP2 was expressed in E. coli and purified as described [40].

### Surface plasmon resonance analysis

The binding between CV2, Chordin and/or BMP2 was recorded on a BIAcore 2000 system (GE-Healthcare, Biosensor) as described [40], [41]. The dissociation constant *K_D_* was calculated from kinetic constants (*k_off_/k_on_*). Per experiment, constants were determined with 6 to 9 different concentrations of the analytes. Mean *K_D_* values and their standard deviations (SD) were calculated from the values of at least three independent experiments. Standard deviations were always below 50%.

### Co-immunoprecipitation (Co-IP)

Co-IPs were performed as described [11], [12]. For Figure 5B, recombinant His_6_-tagged Chordin and His_6_-tagged CV2-T5P/I21R or His_6_-tagged CV2 ΔClip were mixed and incubated in 200 µl binding buffer for 1h at 4°C, followed by BMP2. After incubation at 4°C for 1h, 2.5 µg anti-His-tag antibody (Invitrogen) was added. The complex precipitated with 20 µl protein A Sepharose beads (GE-Healthcare) was split into two parts and subjected to SDS-polyacrylamide gel electrophoresis (SDS-PAGE) under reducing conditions and blotted onto a nitrocellulose filter. BMP2 was detected with an anti-BMP2 monoclonal antibody (R&D Systems), and Chordin and CV2 proteins with the anti-His antibody (Invitrogen).

### Zebrafish experiments

For mRNA in vitro synthesis, the cDNAs of zebrafish CV2, its various fragments, Chordin, Tolloid, Bmp1a and Xenopus TsgW67G were cloned into pCS2+ or pCS2+6×Myc vector [42], followed by linearization of the plasmids with *Not*I and mRNA synthesis, using the Message Machine kit (Ambion, Austin, TX). *bmp2b* mRNA was synthesized as described [43]. Antisense morpholino oligonucleotides (MOs) were as described: *cv2* MO [35]; *chordin* (*chd*) MO [44]; *tolloid* (*tll*) MO and *bmp1a* MO [36]; *sizzled* (*szz*) MO [36]; *noggin1* (*nog1*) MO [45]. mRNAs and MOs were injected into embryos of the 1–4 cell stage as described (1 nl per embryo) [46]. Amounts of reagents injected per embryo are indicated in the corresponding figures. Obtained dorsalized and ventralized phenotypes of injected embryos were determined and classified (V4-C5) at 32 hpf as described [43], [47]. Whole mount in situ hybridization was carried out as described [48], using *eve1* [49] or *otx2* probes [50].

For the detection of Myc-tagged Chordin protein in vivo (Figure 4P), injected embryos were collected at 9 hpf (85% epiboly stage) and homogenized with a pestle in chilled CSH lysis buffer (50 mM Tris-HCl (pH 7.5), 250 mM NaCl, 1 mM EDTA, 0,1% Triton-X100, 1% NP40 and 1× protease inhibitor cocktail; Roche). After centrifugation, supernatants were collected, split into two parts and subjected to immunoprecipitation by incubation at 4°C overnight with 1 µg of 9E10 anti-Myc antibody (Santa Cruz) or 1 µg of anti-GFP antibody (Invitrogen) and 20 µl protein G-Sepharose 4 fast flow (GE Healthcare). The immunoprecipitates were washed three times with PBS, separated via SDS-PAGE, blotted onto nitrocellulose filter and subjected to immunodetection with HRP-conjugated anti-Myc antibody or rabbit anti-GFP antibody followed by HRP-conjugated secondary antibody.
